# Association between Sports-Related Concussion and Mouthguard Use among College Sports Players: A Case-Control Study Based on Propensity Score Matching

**DOI:** 10.3390/ijerph17124493

**Published:** 2020-06-22

**Authors:** Yoshiaki Ono, Yuto Tanaka, Kazuki Sako, Masahiro Tanaka, Junya Fujimoto

**Affiliations:** 1 Department of Special Care Dentistry, Osaka Dental University Hospital, 1-5-17, Otemae, Chuo, Osaka 570-0008, Japan; yoshiaki@cc.osaka-dent.ac.jp; 2 Department of Fixed Prosthodontics and Occlusion, Osaka Dental University, Osaka 540-0008, Japan; sako-k@cc.osaka-dent.ac.jp (K.S.); tanaka-m@cc.osaka-dent.ac.jp (M.T.); 3 Department of Health and Sport Management, Osaka University of Health and Sport Sciences, Osaka 590-0496, Japan; fujimoto@ouhs.ac.jp

**Keywords:** concussion, mouthguard, propensity score

## Abstract

Sports-related concussion (SRC) is a major public health concern. This study aimed to assess the association between mouthguard use and the incidence of SRC in college students through a case-control study using propensity score matching. In total, 195 of 2185 potential participants volunteered to participate in this study. We used Google Forms online to capture participants’ information, including: age; gender; height; weight; sports contact level; level of play; exposure time; frequency of mouthguard use; mouthguard type; and SRC experience. Data for 115 participants who played collision and contact sports were used for the analysis. The difference in the frequency of mouthguard use was assessed between matched pairs and the overall association between SRC and mouthguard use was evaluated. In the matched groups, those who had not experienced SRC wore a mouthguard more frequently than those who had experienced SRC (7/28 vs. 1/28; *p* = 0.051). Logistic regression analysis showed there was a significant negative association between the frequency of mouthguard use and the incidence of SRC (odds ratio 0.101; *p* = 0.041). Within the limitations of this study, these results suggest that mouthguard use may offer some benefit in preventing SRC.

## 1. Introduction

Sports-related concussion (SRC) is a major public health concern [1] for two main reasons. First, SRC occurs frequently, especially in contact and collision sports. For example, a US epidemiological study reported there were approximately 2.8 million traumatic brain injury-related emergency department visits, hospitalizations, and deaths in 2013, including 2.5 million emergency department visits, 280,000 hospitalizations, and nearly 56,000 deaths [2]. Second, excessive biomechanical force to the brain, such as concussion, leads to a neurochemical cascade that affects overall brain function and negatively impacts quality of life [3,4]. For example, concussion has been reported to result in ionic flux, meaning depolarization could then trigger voltage or ligand-gated ion channels, creating a diffuse “spreading depression-like” state [4]. These studies provide evidence that prevention strategies are needed to reduce the number and severity of SRC cases in many sports.

Mouthguards are thought to reduce the risk for SRC because of their shock absorption capability; however, there is mixed evidence that mouthguard use prevents SRC [5,6,7,8,9,10,11,12,13,14,15,16]. A systematic review that analyzed four studies that evaluated the effectiveness of mouthguards in preventing SRCs [9] found the studies reported inconsistent results. One study found that mouthguard use appeared to be associated with a reduced incidence of SRC and loss of consciousness [6], whereas the remaining studies found no beneficial effects of mouthguard use in preventing SRC. A later systematic review analyzed five studies that evaluated the benefit of mouthguard use and revealed a negative incident rate ratio (0.81) against the incidence of SRC, although this was not statistically significant (*p* = 0.18) [15].

A nested cohort study reported that mouthguard use was associated with reduced odds of SRC [16], which was inconsistent with the previous systematic review. A field survey also revealed that wearing a custom-made, properly fitted mouthguard statistically reduced the incidence of SRC when compared with an over-the-counter mouthguard [14]. Therefore, although previous studies have suggested a nonsignificant trend toward a protective effect of mouthguards in collision sports, rigorous case-control designs are required to further evaluate this finding.

We conducted a case-control study to accurately evaluate the association between mouthguard use and risk for SRC by minimizing confounding factors as follows. First, we selected college students who majored in sports and played sport regularly as the study population, which reduced individual differences. Second, we conducted a matched pair analysis based on the propensity score (likeliness of concussion incidence), and then evaluated the relationship between frequency of mouthguard use and history of concussion between the matched groups. Third, we performed a logistic regression analysis by combining multiple factors into a single factor as a propensity score to control participants’ characteristics.

## 2. Methods

### 2.1. Study Population

We invited 2185 college students who majored in sports at Osaka University of Health and Sport Sciences and played sport regularly to participate in the study. Sports types included collision and contact sports (American football, rugby, lacrosse, karate, Nippon kempo, soccer, basketball, handball), limited contact sports (baseball, field hockey, volleyball, kendo), and noncontact sports (soft tennis, softball, lifesaving, swimming, ultimate, track and field). In total, 115 participants played collision or contact sports and their data were used for analysis. This study was conducted with the approval of the Ethics Committee of Osaka Dental University (Approval no. 110986). This study was designed, executed, and analyzed with consideration of the principles outlined in the Strengthening the Reporting of Observational Studies in Epidemiology Statement (STROBE) [17].

### 2.2. Survey Instrument

The survey was administered online through a mobile phone invitation. We created two separate forms. The first form collected participants’ informed consent. The purpose of this study and protection of participants’ personal information were also explained in this form. Participants who read and understood the informed consent form accessed the questionnaire by clicking a link stating “Yes, I read and understood the informed consent form and agree to participate in this survey”. The second form was a questionnaire developed by the Japanese Academy of Sports Dentistry [18]; the primary outcome was changed to SRC and the survey was modified for the Google Forms platform. Briefly, the questionnaire comprised the following questions. Q1-1 How often do you practice each week (days/week)? Q1-2 How long do you practice each day (hours/day)? Q1-3 How many times did you play this game? Q2-1 What type of mouthguard do you use (custom-made/over-the-counter)? Q2-2 How often do you use the mouthguard? Q3-1 Have you suffered SRC during sport? Q3-2 Were you using the mouthguard at the time of SRC?

If a participant clicked “No, I do NOT agree to participate in this survey”, the survey ended. Therefore, we confirmed that participation was voluntary. Participants could not be identified from the information obtained and no plausible harm to participating individuals could arise from the study.

### 2.3. Procedure

This survey was initiated on 9 January 2020 and investigated a 1-year period (1 January to 31 December 2019). The instruction “Please answer the questionnaire for the past year (1 January 2019 to 31 December 2019)” was provided at the beginning of the questionnaire. Data collection finished on 1 March 2020. The questionnaire captured information including age, gender, type of sports, team position, years of competition (i.e., how long the participant had been playing their sport), height, weight, academic year (Japanese university requires 4 years), sports contact level, level of play, exposure time (i.e., practice time in hours per week), frequency of mouthguard use during practice time (none, rarely, often, or every time), type of mouthguard (custom-made or over-the-counter), number of SRCs experienced, and use of a mouthguard at the time of the SRC. Sports contact levels were categorized as “non-contact”, “limited contact”, and “contact or collision”. Level of play was categorized based on participants’ experience in their sport: local, intercollege, and national games.

Participants who played contact or collision sports were included in the present analysis. Frequency of mouthguard use was divided into two subgroups (“none or rarely” and “often or every time”) for statistical analysis, because the numbers of participants who reported they used a mouthguard “rarely” and “often” were small (Table 1).

### 2.4. Statistical Analysis

A propensity score refers to the predicted probability and enables the effects of multiple confounding factors to be calculated as a single score. Therefore, the propensity score is used for two purposes. First, paired subjects matched one-to-one based on the propensity score are comparable or “exchangeable”, which achieves quasirandomization for a simple observational study. Second, the propensity score enabled us to conduct a multivariate analysis with a small sample size. Multiple confounding factors (e.g., age, sex, weight, level of play, and exposure time) can be combined into one propensity score. A multivariate analysis generally requires at least 10–20 samples for an independent parameter. However, use of the propensity score meant that the present study did not require such a large sample size. In this study, a propensity score—the predicted probability that a participant reports the experience of SRC—was obtained for each participant using a logistic regression model [19,20].

The predictor variables for the model included age, sex, weight, level of play, exposure time, and whether the sport required a helmet to be worn. Participants who had experienced SRC were then matched one-to-one, without replacement of those who had not experienced SRC, based on the propensity score using a caliper width equal to 0.2 times the standard deviation of the logit of the propensity score. The difference in the frequency of mouthguard use between the matched pairs was assessed using a chi-square test.

Another unconditional logistic regression analysis was conducted to calculate the odds ratio and 95% confidence interval (CI) for the frequency of mouthguard use against the occurrence of SRC. The occurrence of SRC was considered to be a dichotomous dependent variable. The propensity score was used as a covariate to simultaneously control for other confounding factors such as age, sex, weight, level of play, exposure time, and whether the sport required a helmet. The statistical analyses were performed using R version 3.3.0 (www.r-project.org, R Foundation for Statistical Computing, Vienna, Austria) at a 5% significance level.

## 3. Results

### 3.1. Participants’ Characteristics

Of 2185 potential participants, 195 volunteered to participate in the present study; 197 students accessed the survey (9.0% response rate), but two did not agree to participate. Participants’ demographic data (age, gender, height, weight, body mass index, level of play, exposure time, frequency of mouthguard use, and type of mouthguard) are summarized in Table 1. In total, 115 out of 195 participants played collision or contact sports. These participants were included in the statistical analyses in this study.

### 3.2. Mouthguard Use and Type

Twenty-one participants had their own mouthguard and 94 did not. Fourteen participants used a custom-made mouthguard; of these, 10 used it every time, one often used it, two rarely used it, and one never used it during practice sessions. Seven participants used an over-the-counter mouthguard; of these, five used it every time, one often used it, and one never used it during practice sessions. Ten custom-made mouthguard users suffered five SRCs, and seven over-the-counter mouthguard users suffered one SRC.

### 3.3. Type of Sports and Incidence of SRC

There were three SRCs reported in 12 American football players, 14 in 27 soccer players, 10 in 37 basketball players, three in 25 handball players, two in two rugby players, one in one karate player, and five in two Nippon kempo players.

### 3.4. Relationship between Frequency of Mouthguard Use and Incidence of SRC

Twenty-eight participants had experienced SRC within the previous year, with a total of 35 incidences of SRC. Thirty-one SRCs occurred when a mouthguard was not worn, and four SRCs occurred when a mouthguard was worn.

Propensity score matching resulted in the matching of 28 participants in each group based on their history of SRC within the previous year. Participants’ demographic characteristics in the matched groups were well matched after propensity score matching (Table 2). In the matched group, we compared those who had experienced SRC and those who had not experienced SRC; those who had not experienced SRC wore a mouthguard more frequently than those who had experienced SRC (7/28 vs. 1/28, *p* = 0.051; Table 2).

The logistic regression analysis showed that after controlling for other variables, there was a significant negative association between the frequency of mouthguard use and the incidence of SRC (odds ratio 0.10; 95% CI 0.01–0.91, *p* = 0.041).

## 4. Discussion

This study aimed to document the preventive effects of mouthguard use on SRC. Our analysis revealed that more SRCs occurred when mouthguards were not used during practice sessions than when mouthguards were used. Additionally, participants who had not experienced SRC wore a mouthguard more frequently than those who had experienced SRC (Table 2). Furthermore, our analysis revealed a significant negative association between mouthguard use and the incidence of SRC. These results suggest that mouthguard use may have some benefit in preventing SRC.

We identified a possible reason for the mixed evidence on the association between mouthguard use and SRC. The occurrence of SRC is intrinsically complex. Therefore, a multivariate analysis that considers multiple factors should be conducted. However, such an analysis requires a large sample size, which makes epidemiological research on SRC difficult. Additionally, there are ethical challenges in performing this type of study. For example, it would be unethical to allocate participants to a control group in which they were not allowed to use a mouthguard, as this could put them at greater risk for SRC. Therefore, to address these issues, the Japanese Academy of Sports Dentistry developed a questionnaire to capture all requisite information in a format that could be statistically evaluated to facilitate quantitative analysis of mouthguard efficacy [18]. These data allowed inclusion of participants in each group that did not use a mouthguard as controls, because these participants chose not to use a mouthguard of their own volition.

Unlike several previous systematic reviews, some recent studies have revealed the benefit of mouthguard use in preventing SRC. Winters et al. conducted a prospective study that found wearing a custom-made, properly fitted mouthguard with ≥3 mm thicknesses in the posterior occlusal area statistically reduced the incidence of SRC when compared with over-the-counter mouthguards. That study was superior to similar studies because the quality of mouthguards was strictly controlled and SRC injury was documented by a certified athletic trainer rather than through self-reported data.

Various experimental studies have been conducted to investigate how mouthguards lower the risk for SRC and confirm the efficacy of mouthguards. Hickey et al. revealed that if a mouthguard was in a place in an intact male cadaver and a blow was delivered to the inferior border of the chin, there was a reduction in the amplitude of the intracranial pressure wave and a moderate decrease in the deformation of the cranial bones [21]. In a laboratory study using an artificial skull, Takeda et al. revealed that wearing a mouthguard significantly reduced distortion to the mandibular bone and acceleration of the head when the jaw received a blow from a pendulum compared with not wearing a mouthguard [22,23]. Further, in a human study using a light weight pendulum, Tanaka et al. revealed that wearing a mouthguard reduced the impact to the head during an upward blow to the chin [24]. However, these findings did not provide definitive proof that a mouthguard protects against impact that exceeds the critical threshold of SRC because of ethical reasons. Moreover, a previous epidemiological study revealed that the mechanism of SRC is not only due to a blow to the jaw (1.6%) but is related to different conditions such as impact to the head (74.8%) and contact with ground (11.1%) [8]. Therefore, case-control designs such as the present study that investigated SRCs caused by not only a blow to the mandible but also other conditions using matched pairs (cases: those who experienced SRC; controls: those who did not) analysis based on propensity scores have advantages over previous studies that focused on a limited mechanism of injury.

The frequency of SRCs varied with the type of sport. For example, Nippon kempo is a traditional Japanese full-contact, competitive sparring sport practiced on mats, and players wear a specialized form of armor comprising headgear and breast plate. This seems to be somewhat safer than full contact karate and boxing, which are conducted without protective armor. However, we found five incidences of SRC among two Nippon kempo players in this study. Although our sample size was small, this suggested that serious caution may be required around traumatic brain injuries in Nippon kempo, including SRCs. This may be explained by players being allowed to hit their opponent’s face because armor is used; even if players avoid acute damage, repeated exercises can result in accumulation of brain damage. A possible false sense of security from wearing armor may also mean that players do not defend against potential damage to the brain.

Soccer and basketball also presented higher frequencies of SRC (14 SRCs in 27 soccer players, 10 in 37 basketball players). In Japan, mouthguard use, although recommended, is not mandatory in many sports (including basketball and soccer) unlike in rugby and American football. Implementation of education and enforcement of rule strategies also differ among types of sports. For example, SRC management education programs and sideline SRC check systems are well-prepared in collision sports such as rugby [25]. Therefore, collision and contact sports other than American football and rugby may require more serious SRC prevention strategies.

In epidemiology and public health, it is difficult to conduct randomized experimental studies. Therefore, information about covariates and confounding factors affecting both independent and dependent variables should be used to adjust bias. Propensity score adjustment is widely used in covariate adjustment methods such as matched pair analysis, stratification, and inverse weighting, and it is used as a covariate in applied research [20]. For the present statistical analyses, the propensity score was used for two purposes. First, it was used to create matched pairs and pseudo-randomize participants. The background data of the experimental and control groups could be matched as much as possible instead of conducting a randomized clinical trial. Second, it was used as a covariate in the multivariate analysis. Multivariate analysis used for association tests involving multiple factors requires a large sample size (each factor requires at least 10–20 participants). In this study, the number of participants who experienced SRC was small, and it was determined that the sample size was not sufficient to withstand normal multivariate analysis. Therefore, it was appropriate to perform logistic regression analysis by combining multiple factors into one factor as a propensity score.

Previous studies revealed that mouthguards are a core component in preventing orofacial injuries during sporting activities. For example, a meta-analysis that evaluated 256 studies reported that mouthguard users showed a lower rate of orofacial injuries than nonusers (mouthguard users, 7.5% vs. nonusers, 59.5%) [26]. Mouthguard use is mandated in many contact or collision sports; however, there are several high-risk sports that have not mandated or do not enforce use of mouthguards. A previous observational study evaluated knowledge and attitudes about sports-related dental injuries and mouthguard use in young athletes in four different contact sports (water polo, karate, taekwondo, and handball) [27]. That study reported that a higher rate of dental injuries was observed in several sports in which mouthguard use is not mandated, including water polo (18.6%), karate (17.2%), and handball (21.8%), compared with taekwondo (where mouthguard use is mandated) (3.5%) (*p* = 0.035) [27]. In addition, our previous study conducted among college rugby players revealed that although we required all participants to use their mouthguard, some chose not to use a mouthguard of their own volition during their personal training sessions (the use of mouthguards is mandated during game play) [18]. These findings suggested that education, discipline about the use of protection equipment, and strategies to prevent orofacial injuries are important. Similar strategies can be used to help prevent concussion, especially as there is increasing evidence for the preventive effect of mouthguards on concussion.

The present study had several limitations. With the data available for the multivariate analysis in this study, it was not possible to determine whether the SRC occurred during mouthguard use. However, our comparison of the number of SRCs among participants who did/did not use a mouthguard aimed to overcome this limitation. Participants were explicitly asked whether they had ever suffered a SRC, and if so, whether they were wearing a mouthguard at the time. As all our data were self-reported by participants, it was important to consider whether deficiencies in participants’ recall might have introduced error. For example, our results might have been affected by recall bias wherein one group recalled events more accurately than the other. Additionally, participants who suffered SRC might have had better recall than those who did not, because the SRC sustained could have been more memorable. Additionally, as our response rate was low (9.0%), our results may not accurately represent the characteristics of the whole cohort, and careful interpretation is needed. The small sample size created the following problem. When selected samples are representative, generalization may be possible. A high nonresponse rate is thought to be a causal factor leading to greater nonresponse bias. However, recent studies revealed that a high nonresponse rate does not necessarily equate to greater bias and that the bias may be greater with an arbitrary increase in the response rate [28,29]. Therefore, we believe that despite our low response rate, our results warranted analysis. However, to reduce nonresponse bias, investigators are required to consider the characteristics that influence the response tendency. In our study, we selected college students who majored in sports and played sport regularly as the study population. Participants could easily access the online questionnaire because we posted the questionnaire to the mailing list that all students regularly use to communicate about important matters. Therefore, we believe that the low response rate in our study might not have resulted in bias.

Furthermore, our results should be interpreted with caution when it comes to sports requiring protective equipment other than mouthguards. For example, American football and Nippon kempo require headgear, whereas other collision and contact sports such as soccer and basketball do not. A previous systematic review revealed that helmet use reduced head injury risk [12]. Additionally, the incidence of concussion depends on the acceleration acting on the head and the frequency of the strokes. These values were not evaluated in our study. However, obtaining such values is difficult because it requires constant surveillance of participants by the investigators.

## 5. Conclusions

Our study revealed three main findings. First, more SRCs occurred when mouthguards were not used. Second, those who had not experienced SRC wore a mouthguard more frequently than those who had experienced SRC. Third, our analysis also revealed a significant negative association between mouthguard use and the incidence of SRC. Within the limitations of this study, these results may suggest that mouthguard use helps prevent SRC. However, these conclusions do not necessarily suggest that wearing a mouthguard provides sufficient protection against concussion, and mouthguards should not be considered a replacement for other protective headgear.

## Figures and Tables

**Table 1:** Characteristics of the study participants (*n* = 115).

Characteristics	Participants Who Have a Mouthguard (*n* = 21)	Participants Who Do Not Have a Mouthguard (*n* = 94)
Age (y), mean ± SD	20.5 ± 1.1	19.9 ± 1.0
Gender, *n*		
Female	9	27
Male	12	67
Height (cm), mean ± SD	171.6 ± 10.9	171.9 ± 8.5
Weight (kg), mean ± SD	69.7 ± 14.1	68.7 ± 10.8
Body mass index (kg/m^2^), mean ± SD	23.4 ± 3.0	22.6 ± 1.7
Level of play, *n*		
No experience	2	16
Local games	5	26
Intercollege games	8	30
National games	6	22
Exposure time (hours/week), mean ± SD	20.6 ± 11.8	18.2 ± 8.5
Frequency of mouthguard use, *n*		
Every time	15	
Often	2	
Rarely	2	
Never	2	
Type of mouthguard, *n*		
Custom-made	14	
Over-the-counter	7	
A number of those who experienced concussion, *n*		
Two or more	2	8
One	2	20
None	17	66
Type of sport, *n*		
American football	11	1
Basketball	0	37
Handball	0	25
Karate	1	0
Lacrosse	7	1
Nippon kempo	0	2
Rugby	2	0
Soccer	0	28

SD, standard deviation.

**Table 2:** Participants’ demographic characteristics and history of concussion within the previous year after propensity score matching (*n* = 56).

Characteristic	Concussion (−) (*n* = 28)	Concussion (+) (*n* = 28)	*p*
Age (y), mean ± SD	19.6 ± 0.9	20.0 ± 1.0	0.11
Female, *n*	7	7	1
Weight (kg), mean ± SD	71.6 ± 10.9	73.0 ± 11.6	0.64
Exposure time (hours/week), mean ± SD	19.5 ± 13.8	23.5 ± 18.2	0.37
Custom-made mouthguard users, *n*	8	7	1
Level of play, mean ± SD	1.4 ± 1.0	1.6 ± 1.1	0.54
Wearing a helmet required	4	4	1
Frequency of mouthguard use, *n*			
None or rarely	21	27	0.051
Often or Every time	7	1	

*t*-test used for the continuous variables, and chi square test used for categorical variables. SD, standard deviation. Level of play categorized based on participants’ experience of local games = 0, intercollege games = 1, and national games = 2. Whether the sport required a helmet to be worn was categorized based on sports where wearing a helmet is required (American football, rugby, lacrosse, Nippon kempo) = 1; sports where a helmet is not required (karate, soccer, basketball, handball) = 0.
